# The Development and Inclusion of Questions on Surgery in the 2018 Zambia Demographic and Health Survey

**DOI:** 10.9745/GHSP-D-20-00619

**Published:** 2021-12-31

**Authors:** Sabrina Juran, Sanna Moren, Vatshalan Santhirapala, Lina Roa, Emmanuel Makasa, Justine Davies, Jose Miguel Guzman, Lars Hagander, Hampus Holmer, Mark G. Shrime, Thomas G. Weiser, John G. Meara, Stefanie J. Klug, David Ljungman

**Affiliations:** a Program in Global Surgery and Social Change, Harvard Medical School, Boston, MA, USA.; b Epidemiology, Department of Sport and Health Sciences, Technical University Munich, Germany.; c Department of Surgery, Institute of Clinical Sciences, Sahlgrenska Academy, University of Gothenburg, Sweden.; d Region Västra Götaland, Sahlgrenska University Hospital, Department of Surgery, Gothenburg, Sweden.; e Department of Obstetrics & Gynecology, University of Alberta, Edmonton, Canada.; f Wits Centre of Surgical Care for Primary Health and Sustainable Development, Faculty of Health Sciences, University of Witwatersrand, Johannesburg, South Africa.; g MRC/Wits Rural Public Health and Health Transitions Research Unit, School of Public Health, Faculty of Health Sciences, University of Witwatersrand, Johannesburg, South Africa.; h Centre for Global Surgery, Department of Global Health, Stellenbosch University, Cape Town, South Africa.; i Institute of Applied Health Research, University of Birmingham, United Kingdom.; j NoBrainerData, Columbia, MD, USA.; k WHO Collaborating Centre on Surgery and Public Health, Pediatric Surgery, Department of Clinical Sciences in Lund, Faculty of Medicine, Lund University, Lund, Sweden.; l Department of Global Public Health, Karolinska Institutet, Stockholm, Sweden.; m Institute of Global Surgery, Royal College of Surgeons in Ireland, Dublin, Ireland.; n Department of Surgery, Stanford University, Stanford, CA, USA.; o Department of Clinical Surgery, University of Edinburgh, Edinburgh, United Kingdom.; p Department of Plastic and Oral Surgery, Boston Children's Hospital, Boston, MA, USA.

## Abstract

**Background:**

While primary data on the unmet need for surgery in low- and middle-income countries is lacking, household surveys could provide an entry point to collect such data. We describe the first development and inclusion of questions on surgery in a nationally representative Demographic and Health Survey (DHS) in Zambia.

**Method:**

Questions regarding surgical conditions were developed through an iterative consultative process and integrated into the rollout of the DHS survey in Zambia in 2018 and administered to a nationwide sample survey of eligible women aged 15–49 years and men aged 15–59 years.

**Results:**

In total, 7 questions covering 4 themes of service delivery, diagnosed burden of surgical disease, access to care, and quality of care were added. The questions were administered across 12,831 households (13,683 women aged 15–49 years and 12,132 men aged 15–59 years). Results showed that approximately 5% of women and 2% of men had undergone an operation in the past 5 years. Among women, cesarean delivery was the most common surgery; circumcision was the most common procedure among men. In the past 5 years, an estimated 0.61% of the population had been told by a health care worker that they might need surgery, and of this group, 35% had undergone the relevant procedure.

**Conclusion:**

For the first time, questions on surgery have been included in a nationwide DHS. We have shown that it is feasible to integrate these questions into a large-scale survey to provide insight into surgical needs at a national level. Based on the DHS design and implementation mechanisms, a country interested in including a set of questions like the one included in Zambia, could replicate this data collection in other settings, which provides an opportunity for systematic collection of comparable surgical data, a vital role in surgical health care system strengthening.

## Abstract (precis)

Data from household surveys serve as the backbone to sustainable development planning. For the first time, questions on surgery have been included in a nationwide Demographic and Health Survey, showing that it is feasible to integrate these questions into a large-scale survey.

## Teaser

**Key Findings:**

- Based on this pilot survey, which responded to the call to collect, aggregate, and analyze global anesthesia and surgery data, we conclude that it is feasible to integrate surgical questions into a large-scale sample survey to provide insight into surgical needs at a national level.
- Approximately 5% of women and 2% of men had undergone an operation in Zambia in the past 5 years. Among women, cesarean delivery was the most common surgery, while circumcision was the most common procedure among men.
- The diagnosed unmet need for surgical care in Zambia is estimated to be 65%.

**Key Implications:**

- For health system strengthening, baseline data and monitoring of progress are necessary. Improved data capabilities allow for mapping out and understanding systems.
- Data collection mechanisms pertinent to surgical care need to become part of routine data collection processes.
- There is a need to build on existing data collection mechanisms to avoid adding unnecessary burden, information overload, and data collection fatigue.
- The replicable design of the Demographic and Health Survey allows for the systematic collection of comparable surgical data in other settings. Similar questions could be included in other surveys as well.
- There is a need to establish data pathways to ensure clear responsibilities among national and international institutions and integrate surgical metrics into existing mechanisms for sustainable data collection.

## INTRODUCTION

Globally, it is estimated that 5 billion people do not have access to essential surgical, obstetric, and anesthesia care.^1^ Scaling up access to surgery, obstetrics, and anesthesia health care services is key to accelerating progress toward the SDGs.^2^ The 2015 World Health Assembly Resolution 68.15^3^ and Decision A70.22 ^4^ recognize the role of surgical and anesthesia health care for universal health coverage (UHC) and the importance of surgery and anesthesia as crosscutting treatment modalities in resilient health systems. Furthermore, the World Health Organization (WHO) along with the 3^rd^ edition of the *Disease Control Priorities* recommend basic surgical health care to the essential packages of interventions delivered at first-level hospitals in low- and middle-income countries (LMICs).^5^^,^^6^ Yet, many LMICs continue to have a large unmet need for surgical health care,^7^^–^^12^ while most public health data collection tools remain blind to the problem.

To achieve ambitious goals and strengthen health systems to ensure no one is left behind, data are needed to track progress. The Lancet Commission on Global Surgery developed 6 indicators for data collection and developed methods to create standardized and comparable datasets across countries over time. These indicators assess the preparedness, delivery, and impact of surgical health care.^1^ Some of these indicators have been recognized as World Bank Development Indicators,^13^ and all 6 are included in the WHO list of 100 core health indicators.^14^ However, the availability of data regarding surgical, anesthesia, and obstetric care remains low.^15^ To become part of routine data collection processes, it is necessary to establish nationally driven sustainable data collection mechanisms pertinent to surgical care. Meanwhile, in light of demands being placed on national statistical systems to report numerous development indicators, there is a need to build on existing data collection mechanisms to avoid adding unnecessary burden, information overload, and data collection fatigue. This would include other household surveys, such as Multiple Indicator Cluster Surveys, and facility-based tools (e.g., District Health Information System, Service Provision Assessment questionnaire, and the Service Availability and Readiness Assessment).

Household surveys are considered the most reliable data source on individual knowledge, attitude, and practice, as well as critical determinants of health status and health usage.^16^ Household surveys can fill crucial gaps, assessing the need for surgical care and providing data on the main barriers to meet such needs as limited evidence exists on the barriers to access surgical and anesthesia care.^17^^,^^18^

Household surveys can fill crucial gaps, assessing the need for surgical care and providing data on the main barriers to meet such needs as limited evidence exists on the barriers to access surgical and anesthesia care.

The Republic of Zambia has taken a leadership role in promoting surgical and anesthesia health care as an essential component of UHC. The Zambian Ministry of Health (MOH) prioritized the development and costing of a National Surgical Plan (2017–2021) launched at the World Health Assembly in 2016 and fully integrated into the Zambian National Health Strategic Plan 2017–2021.^19^^,^^20^

The 2018 Zambia Demographic and Health Survey (ZDHS)^21^ was implemented by the Zambia Statistics Agency (ZamStats) in collaboration with the MOH. It was financed by the United States Agency for International Development (USAID), the Department for International Development of the United Kingdom, and the Global Fund. Due to Zambia's commitment to advance surgical health care, this survey offered an opportunity to explore the addition of questions addressing surgical needs. Under the leadership of the Zambian MOH, it was decided to develop a short set of questions that could be added to this survey and would show the feasibility of using household surveys to collect data on access to surgical care. Whereas the National Surgical Plan was based on modeled and pre-existing data, implementation of surgical questions in ZDHS provides for systematic, continuous, and reproducible data collection, yielding more reliable, nationally representative, and comparable results. The ZDHS, although collecting fewer data points, is nationally representative. Local leadership is a prerequisite for ownership, in-depth engagement of local stakeholders around surgical systems strengthening, and national health policy planning.^22^^,^^23^

We aimed to describe the design of a set of surgical questions for DHS and their integration into the 2018 ZDHS. We also present preliminary results from these questions, with the intent to provide insight into the utility of ZDHS to collect data on the perceived need for, and access to, surgical care in Zambia.

## METHODS

USAID has been implementing the DHS Program for more than 30 years, collecting and disseminating data on population and health through more than 400 surveys in over 90 countries since 1984. DHS data are gathered through nationally representative, repeated household surveys, using standardized questionnaires and enables comparison between and across countries.^24^ Questionnaires consist of a core module and a series of elective modules that countries select from. There are 5 standard model questionnaires; man's, woman's, household, fieldworker, and biomarker questionnaire, where elective modules can be added, such as the survey on maternal mortality.^25^

### Surgery Question Design

Based on matching of variables and classification systems of existing data collection mechanisms, the authors identified data sources for surgical statistics, including facility-based data as well as population-based approaches. As a population-based approach, household surveys were considered advantageous as they can reach those who otherwise would not have access to or have not tried to reach surgical care.

Questions on access to surgical, anesthesia, and obstetric care were designed for addition to the female and male DHS questionnaires planned for roll out in Zambia. Questions were designed through an iterative consultation process during 2017 and 2018, involving an expert panel from the surgical research community with a research focus on global surgical data. The 13 group members (SJ, SM, VS, LR, JD, MS, TW, LH, HH, EM, JGM, JMG, DL) from Africa, Europe, North America, and South America brought together expertise from the fields of medicine, epidemiology, statistics, and demography. The expert group proposed a longer list of questions, while statisticians from the ZamStats provided guidance on the selection of the final set of questions to prioritize the inclusion of locally relevant surgical health care metrics and avoid information overload and data collection fatigue. Adhering to the DHS questionnaire format, the questions were fine-tuned for clarity, comprehensibility, and utility.

The consultation process reached consensus on 5 topics representing 4 central themes:

1. Surgical volume (service delivery)
2. Types of surgery (service delivery)
3. Diagnosed need for surgical health care (diagnosed burden of surgical disease)
4. Barriers to accessing surgical health care (access to care)
5. Maternal mortality (quality of care)

These topics were used as a basis to construct a set of nonambiguous questions with good face validity.

### Surgical Volume (Service Delivery)

The Lancet Commission on Global Surgery estimates an operative volume of 5,000 surgical cases per 100,000 population as a minimum threshold target. The first question aimed at generating an estimate of surgical volume and an output measure of service delivery by asking: “Have you ever undergone a surgical operation in the past 5 years?”

The overall definition of surgical procedure applied in this work follows that of the Lancet Commission of Global Surgery, which is a procedure performed in an operating room under any kind of anesthesia. The timeframe of 5 years was deemed an ideal between achieving adequate power without risking substantial recall bias.

### Types of Surgery (Service Delivery)

To establish the met need of surgery by broad categories of procedures, the next question asked respondents that had undergone surgery in the past 5 years, “What type of operation(s) were they?” Response options were provided from a spectrum of common surgical procedures, and several responses could be recorded for each subject (Table 1).

**TABLE 1.** Surgery-Related Questions Added to the 2018 Zambia Demographic and Health Survey

Women's and Men's Questionnaire
Have you ever undergone a surgical operation in the past 5 years?
Yes
No
What type of operation(s) were they? (Name all that apply)
Hernia operation
Cesarean delivery (women's questionnaire only)
Hydrocele operation (men's questionnaire only)
Laparotomy (cutting open the abdomen)
Lump removal
Abscess drainage
Wound closure
Open fracture
Other (specify)
In the last 5 years has a doctor or another healthcare worker told you that you might need (another) operation?
Yes
No
Were you able to access it?
Yes
No
Why did you not access it? (Record all mentioned)
I could not reach the doctor
I could not afford the operation
I could not afford to get to the hospital
I could not afford the time off work
It was too far to get to the hospital
I did not trust the operation would make me better
Fear of care
Out of shame
My spouse/family would not let me go
Other (specify)
**Maternal Mortality Module**
Did (name) receive a cesarean delivery?
Did (name) die in the hospital?

The interviewer manual provided explanations for interviewers to use if further clarification regarding procedures were necessary. These procedures were selected because they were considered as common, conceptually accessible for laypeople, and rely on the availability of a wide array of surgical services, providing additional insight about the surgical capacity in the country. Stratification by procedure was adopted as it was considered valuable in providing possible insight for projecting infrastructure and workforce development needs. The inclusion of cesarean delivery, laparotomy, and open fracture management are recognized as “Bellwether procedures,” therefore may reflect broader surgical system capacity regionally. During data collection, when looking at the open responses in the option “Other (specify),” ZamStat added and coded the following response variations not considered in the questionnaires:

- Mastectomy (women's questionnaire only)
- Fistula repair (women's questionnaire only)
- Ectopic pregnancy (women's questionnaire only)
- Hysterectomy (women's questionnaire only)
- Female tubal ligation (women's questionnaire only)
- Circumcision (men's questionnaire only)
- Appendicitis
- Minor operation
- Eye surgery

### Diagnosed Need for Surgical Health Care (Diagnosed Burden of Surgical Disease)

The third question is assessing the perceived total demand for surgical health care by asking respondents, “In the last 5 years has a doctor or another health care worker told you that you might need (an/another) operation?” We deemed this question to be a pragmatic proxy for actual need for surgery, validated by a health care worker, that owned some validity rather than simply asking if subjects themselves believed they needed surgery.

### Barriers to Accessing Surgical Health Care (Access to Care)

Unmet need for diagnosed surgical care is defined as the number of individuals who in the last 5 years had been recommended by a doctor or another health care worker that they needed surgery and could or did not access it. Multiple response options to the question, “Why did you not undergo it [the surgery]?” were built upon social, cultural, structural, and financial dimensions as well as the patient's beliefs, views, and expectations. Structural barriers refer to the location of health care facilities and the availability of these facilities to the population, while financial dimensions refer to medical and non-medical costs such as transport and time off work. Here, ZamStat added the response alternative that the surgery was not needed anymore.

### Maternal Mortality (Quality of Care)

Although quality of care is multifaceted and complex to measure, perioperative mortality is considered a baseline indicator of surgical care quality.^26^ Due to the need for case mix and risk factor adjustment for interpretation of this indicator a specific intervention ideally should be chosen. The volume and homogeneity of the cesarean delivery make this an appropriate choice. Also, mortality after cesarean delivery has been recognized in both systematic reviews and prospective observational data^27^ to discriminate strongly on health system quality, with estimates that maternal mortality after cesarean deliveries are 50–100 times higher in LMICs than in high-income countries.^28^

Information on maternal death after cesarean delivery health care may be limited due to the overall low number of cases of maternal death in sampled households, owing to the rarity of maternal deaths. However, the expert panel felt this was an essential quality metric that should be explored in this pilot survey to measure and improve 1 component of the quality of obstetric health care. Quality of maternal health care is an essential health measure, and this may provide critical data for quality improvement.^29^

In the maternal mortality section of the ZDHS survey, respondents were asked about the survival of their siblings. Two questions for all women, relevant for surgical analysis, were included. In the case of sisters having died during childbirth or within 2 months after the end of a pregnancy or childbirth, respondents were asked “Did (name) receive a cesarean delivery?” A second question about that same woman, and/or those women that were pregnant when they died (all maternal deaths), was asked “Did (name) die in the hospital?”

### Data Collection

In parallel to the consultative process of question design, stakeholders within and associated with the Zambia MOH argued for the adoption of the questions regarding surgical care in the 2018 ZDHS. In 2018, questions to collect data on access to timely essential surgery and surgical volume were added to the woman's and man's health questionnaire and the adult and maternal mortality module of the ZDHS. While the woman's questionnaire was used to collect information from women aged 15–49 years; the man's questionnaire was used to collect information from men aged 15–59 years. All permanent residents of a selected household or visitors who stayed in the households the night before the survey were eligible to be interviewed. Data were collected by the implementing organization (ZamStats) and were gathered for the individual interviewed, apart from questions on maternal mortality, which were aimed at sisters of women who had passed during pregnancy, childbirth, or within 42 days of delivery or end of a pregnancy.

After the questionnaires were finalized in English, they were translated into 7 major languages: Bemba, Kaonde, Lozi, Lunda, Luvale, Nyanja, and Tonga. Data collection took place from July 18, 2018, to January 24, 2019, as part of a standard DHS. The sampling frame used for the 2018 ZDHS was the 2010 Population and Housing Census of the Republic of Zambia conducted in 2010 by ZamStats. Twenty-two teams of 7 enumerators set out to gather information from sampled 13,595 households across the Zambian territory.

### Data Analysis

SPSS was used to extract data from 2 datasets containing data from the men's questionnaire and the women's questionnaire, also holding data on adult and maternal mortality, called “men's recode” and “individual recode,” respectively. This has been presented using descriptive frequency analysis with calculations of percentages of the survey population. Results for surgical volume were already disaggregated by sex, age, and place of residence in the ZDHS main report and are presented as such in this text. No missing values were found for the data relevant for surgical care.

## RESULTS

The group reached consensus on adding 5 questions to the women's and men's questionnaire and 2 questions to the maternal health questionnaire (Table 1).

Of the 13,595 households sampled, 12,943 were occupied and 12,831 interviewed, yielding a response rate of 99.1%.^21^ Of these, 4,714 households with urban and 8,117 households with rural residence were interviewed. Please see ZDHS 2018 publication^21^ for full sampling methodology.

### Surgical Volume (Service Delivery)

In the sampled population of 25,830 individuals, 13,683 were women aged 15–49 years and 12,132 were men aged 15–59 years. We found that 631 (4.6%, 95% confidence interval [CI]= 3.0%, 6.2%) women and 230 (1.9%, 95% CI=0.1%, 3.7%) men had undergone at least 1 operation in the last 5 years yielding a total of 861 surgical procedures. Subjects were able to report on having undergone several procedures.

### Type of Surgery (Service Delivery)

The most common surgical procedure overall was cesarean delivery (n=470). Figure 1 presents the other types of surgery with their respective frequencies.

**FIGURE:**
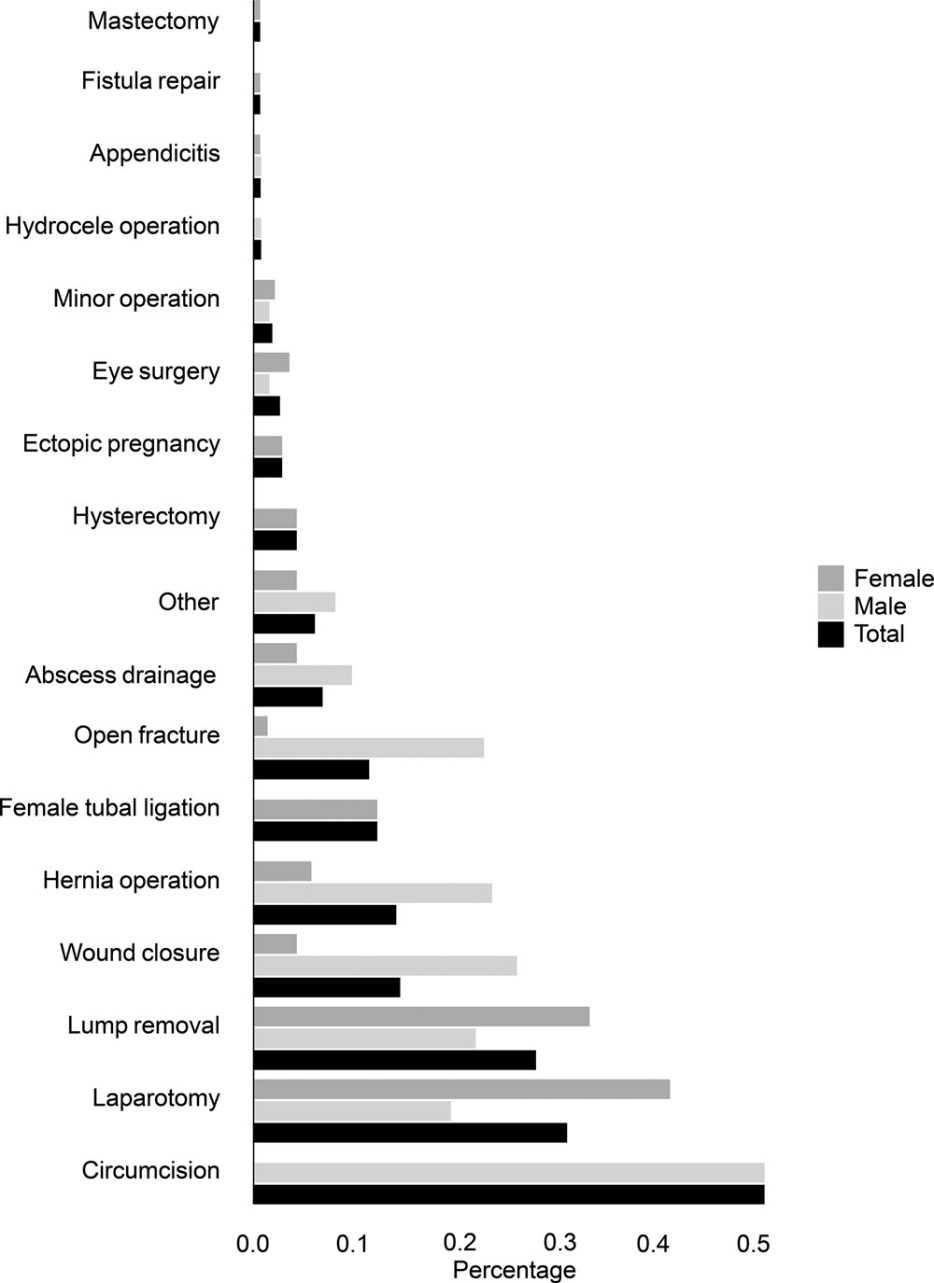
Type of Surgery^a^ Performed in the Last 5 Years Among Women (n=630) and Men (n=230), 2018 Zambia Demographic and Health Survey ^a^Cesarean delivery (3.4% of women) has been omitted, as the frequency was much higher than the second most common procedure.

### Diagnosed Need For Surgical Health Care (Diagnosed Burden of Surgical Disease)

Among all respondents, 157 persons reported that, in the past 5 years, they had been told by a doctor or other health care worker that they might need surgery. Combining these with the 861 individuals that underwent surgery, this suggests a total diagnosed need for surgery of 3.97% (n=1026, 95% CI=2.78%, 5.16%) in the sampled population over the past 5 years.

### Barriers to Accessing Surgical Health Care (Access to Care)

Of the 157 persons who reported having been told they needed surgery, 55 persons reported they had undergone the relevant operation. Based on the previous definition and the numbers ZDHS yields, an estimate of the diagnosed unmet need for surgical care in Zambia is at 65%, where 102 of 157 people did not undergo the surgery which they were recommended. The reasons for not accessing care are listed in Table 2.

**TABLE 2.** Reasons Why 2018 Zambia Demographic and Health Survey Respondents Reported Not Accessing Surgical Health Care in Zambia

	Men	Women	Total	Population, %
Did not trust the operation would make me better	7	8	15	0.06
Fear of care	6	8	14	0.05
Could not reach the doctor	7	7	14	0.05
It was not needed anymore	1	13	14	0.05
Could not afford the operation	4	7	11	0.04
Spouse/family would not let me go	5	5	10	0.04
Could not afford to get to the hospital	3	3	5	0.02
Was too far to get to the hospital	3	1	4	0.02
Could not afford time off work	1	2	3	0.01
Other	4	8	12	0.05
Sum	41	61	102	0.40

### Maternal Mortality (Quality of Care)

In the maternal mortality section of the ZDHS survey, respondents were asked about the survival of their siblings. The results for the respondents' sisters are listed in Table 3.

**TABLE 3.** 2018 Zambia Demographic and Health Survey Respondents' Answers on Maternal Mortality

**Total Number of Female Siblings of Respondents**	**36,114**
Total deaths among female siblings	3,986 (11% of female siblings, 95% CI=10%, 12%)
Total maternal deaths	221 (5.5% of deaths among female siblings, 95% CI=2.5%, 8.6%)
Total cesarean deliveries among maternal deaths	81 (37% of maternal deaths, 95% CI=26%, 47%)
Total maternal deaths in hospital	48 (22% of maternal deaths, 95% CI=10%, 34%)

Abbreviation: CI, confidence interval.

The maternal mortality ratio was calculated for the ZDHS in their publication for the 7-year period before the 2018 ZDHS, as an estimate of 252 deaths per 100,000 live births (CI 158, 357).

## DISCUSSION

The surgical questions were piloted in Zambia with an extremely high response rate of 99.1% displaying limited utilization of surgical services for women (4.6%) and men (1.9%) in the last 5 years. Cesarean delivery constituted nearly 4 of 5 surgical procedures in women.

Of the 157 people who reported having been told they needed surgery, 55 patients had undergone surgery, indicating a met need among diagnosed patients of 35%. The actual need is expected to be largely underestimated due to several barriers to seeking and receiving surgical care. Recognizing the challenge of capturing the true population-based need for surgery, the data coming from this question provide an insight into the need for surgery that was diagnosed by a provider. We recognize that it will underestimate the overall burden of surgical disease, as it excludes all of those who have never been diagnosed. It could be possible to include more subjects by asking about perceived surgical need, but the most relevant information would be achieved by diagnosing and reporting every need.

Recognizing the challenge of capturing the true population-based need for surgery, the data from asking whether they had been told they needed surgery provided insight into the need for surgery that was diagnosed by a provider.

Although 861 people reported having undergone surgery, 157 people reported having been told that they needed an/another operation, and the gap between these 2 still needs to be investigated. The questions regarding surgical volume and diagnosed need for surgical health care as well as barriers to accessing surgical health care were considered to represent different aspects of receiving surgery. Therefore, their results were not combined when calculating diagnosed unmet need.

Fear and mistrust were the most cited reasons for not seeking surgical care despite health provider recommendations. This may indicate that the perceived quality of surgical care is variable in this group of surveyed patients, impacting health care utilization.^30^ Qualitative community-based studies may further elucidate patient perceptions of surgical system performance. To monitor unmet need for surgery as a component of universal health coverage, it is essential to account for people who need but do not access surgery and to ascertain the barriers.

Sample household surveys appear to be the most appropriate source for this. With improved definitions, collection, and aggregation, surgical indicators can play a great role in informing evidence-based health policy to improve access to safe, affordable, and timely surgical, obstetric, and anesthesia health care. Previous data collection on surgical health care has often been 1-time initiatives driven by academic institutions, predominantly from high-income countries^18^ and estimates available on the need for surgical health care in LMICs are frequently modeled based on small sample studies. Existing data are very heterogeneous, making comparison difficult across countries and time.^15^^,^^17^ Further, aggregated data often hide subnational disparities,^31^ hence disaggregated data are needed to investigate the true unmet need of surgical health care and the inequalities in access to health care at the global, national, and subnational levels.

This pilot of integrated questions on surgical health care suggests that the question design proved successful overall. Low surgical volume due to poor access to surgical care may require a longer recall period to obtain sufficient power. Thus, 5 years was considered a golden mean between the 2 trade-offs.

This pilot of integrated questions on surgical health care suggests that the question design proved successful overall.

Cesarean delivery was the most common surgical procedure and represented 4 of 5 surgeries for women. This is consistent with literature where cesarean delivery makes up a large proportion of the surgical volume in countries with very low health expenditure per capita, whereas this has been shown to be a much smaller percentage of 2.7% in high-expenditure countries.^17^^,^^35^ Maternal mortality is a rare event on a population level. The 2 questions related to maternal death after cesarean delivery in ZDHS yield case numbers too low for further analysis. This suggests that, for better resolution, quality of care, in contrast to access to care, primarily ought to be investigated in the population with met need, (i.e., through facility assessments and targeted patient-related outcome measures). One limitation with the structure of interviewing women regarding deaths of sisters is that multiple siblings may have reported on the death of 1. However, this also applies to survivors and is an inherent part of the DHS structure.

The gap in surgical data is concerning, particularly in the poorest countries of the world. The paucity of surgical data globally and the challenges with systematic and reliable data collection have hindered surgical system strengthening.^18^ Incorporating surgical questions into DHS facilitates systematic collection of population-based data that is sustainable, replicable, and comparable. The quality of the data is assured by following up with a set of quality control tables that allows DHS for online and real-time verification of data that is being collected. However, as the information on surgery was collected for the first time in a DHS, there is no reference for comparison.

### Future Directions

Going forward, validation of survey responses with clinical examination and other forms of diagnostic evaluation, such as radiographic testing, in a defined population would be valuable. A potential development would be to integrate sample surveys data with population census data to allow the generation of small area estimates for more granular data.

Future DHS can rely on the lessons learned and data collected in Zambia for quality assessments. Based on the inclusion process of questions in the ZDHS and a successful argument for inclusion of these questions in wave 8 of the DHS, the DHS Program has designed a new module with these questions integrated.

Based on the inclusion process of questions in the ZDHS and a successful argument for inclusion of these questions in wave 8 of the DHS, the DHS Program has designed a new module with these questions integrated.

In addition to DHS, questions on surgical health care could be included in other household surveys such as Multiple Indicator Cluster Surveys. Similarly, facility-based surveys such as the Service Provision Assessment questionnaire and the Service Availability and Readiness Assessment could benefit from the inclusion of questions pertinent to surgery to capture domains of health care not currently captured by facility assessments.

### Limitations

As individuals may have undergone multiple procedures within the 5 years, a limitation may be the challenges to recall that information for several years back. A further limitation is that data from the survey are likely to underestimate unmet need for surgical health care. The 3-delay model on barriers faced by mothers in accessing maternal health care is readily applicable to surgical health care.^32^ The first delay, “delay in seeking health care,” is considered a significant barrier, where a combination of health literacy and access to community health care are key factors. Furthermore, assumptions are made that the health care professionals who screen patients are able and willing to recognize and communicate the need for surgical health care. We recognize that the respondents' responses relied on self-assessments of the patient population, without professional validation of physical conditions, which has been the approach of other similar studies.^33^^,^^34^

## CONCLUSIONS

Questions on surgical health care were successfully developed and implemented in ZDHS. The aims to generate evidence on the perceived prevalence of surgical conditions, a characterization of the diagnosed need for treatment as well as the identification of inequalities and barriers to access were achieved.

The Zambian experience serves as guidance for other countries on how to introduce new questions into existing DHS modules and as an example for other countries to replicate Zambia's efforts to integrate surgical data collection into existing household surveys. The collected data can provide important information on the needs of the population and the surgical system and may be of great use for policy makers and other stakeholders.

## Notes

**Peer Reviewed**
